# Kidneys also "speak Portuguese"

**DOI:** 10.1590/2175-8239-JBN-2020-0264

**Published:** 2021-03-05

**Authors:** Iara da Silva Santos, Maria Júlia Correia Lima Nepomuceno Araújo, Vanda Jorgetti, Rosilene Motta Elias, Jordi Bover

**Affiliations:** 1 Universitat Autònoma de Barcelona, Hospital Germans Trias, Barcelona, España.; 2 Universitat Autònoma de Barcelona, Fundació Puigvert, Barcelona, España.; 3 Hospital Nove de Julho, São Paulo, SP, Brasil.; 4 Hospital das Clínicas, São Paulo, SP, Brasil.

To the Editor:

Recently, the international initiative KDIGO (Kidney Disease: Improving Global Outcomes) published the important conclusion of a consensus conference generically titled "Nomenclature for kidney function and disease", whose main goal was the standardization of nephrological nomenclature used in scientific articles published in English, based on the best understanding by patients (anglo-saxons) as the fundamental principle^1^^,^ 2. As nephrologists, we must take these recommendations into account in our international publications.

However, it was surprising that one of the main recommendations was to use the term "kidney" rather than "renal" for general descriptions of kidney function and disease, assuming that it is easier for patients to comprehend terms incorporating the more familiar adjective "kidney" rather than the Latin adjective "renal"^2^.

Interestingly, this decision does not seem to induce a change in the nomenclature of anatomical structures (renal artery) or historically established names. Besides, also in a logical decision, the Greek prefix "nephron-" was maintained in terms such as nephritic syndrome, nephrotic syndrome, nephropathy etc., as well as the name of our specialty (Nephrology)^2^.

English dominates scientific publications worldwide, corresponding to the language used in 3/4 of all papers. This is somewhat unfair to many non-English-speaking scientists, which often have difficulty in publishing their papers because of the language. Nonetheless, there is a consensus that a unified language is beneficial in making the research globally recognizable. Even in this Journal, from the Brazilian Society of Nephrology, the publications are in both Portuguese, the official language in Brazil, and in English.

In the past, there were times for French, German, and Italian languages' domination in the scientific literature. And while English is the dominating language now, who knows what language will be recommended for science in 100 years? Latin-derived terms such as nephron and nephrology are common in many languages. It is estimated that 700 million people speak Spanish, French, Italian, or Portuguese worldwide. Therefore, it is surprising that we, nephrologists from countries with Latin-based languages, should adopt the terms kidney disease instead of renal disease. We usually refer to "renal" problem instead of a "kidney" problem. If the goal of the consensus was to promote a better understanding, it did not consider a Brazilian patient, for example. Therefore, we believe that this should be taken into consideration for the consensus of global nomenclature.

In agreement with this, a team effort of 10 nephrologists from 9 different countries has proposed to clarify the nomenclature and acronyms in academic publications^3^. We, Brazilians, should join them in suggesting that Latin-based non-Anglo-Saxon terminology should also be considered appropriate. We should not forget that Latin was the language of science in the past, English is the dominant language today, and perhaps in the future Chinese will take the place. Who knows?

Finally, we emphasize that it is not only a matter of "renal" being our natural adjective for kidney-related structures or conditions in the scientific field, but also, in the current clinical scenario, the doctor cannot forget that the patient and treatment individualization play a central role in shared decision-making^4^.
